# Where Do We Go From Here? Defining an Agenda for Home-Based Records Research and Action Considering the 2018 WHO Guidelines

**DOI:** 10.9745/GHSP-D-18-00431

**Published:** 2019-03-22

**Authors:** David W. Brown, Xavier Bosch-Capblanch, Lora Shimp

**Affiliations:** a Brown Consulting Group International LLC, Cornelius, NC, USA.; b Swiss Tropical and Public Health Institute, Basel, Switzerland.; c John Snow Inc, Rosslyn, VA, USA.

## Abstract

Recent WHO guidelines point to knowledge gaps about home-based records despite their widespread use. Future research should explore their impact on health outcomes, challenges including production costs and confidentiality breaches, the role of design in their use, and the business case for investing in them.

Home-based, personal health records—such as vaccination cards or child health passports—are an important public health tool and serve many critical roles, especially in the delivery of immunization services (Box 1). Home-based records (HBRs) provide frontline health care workers with a standardized patient history that is convenient, comprehensive, and vital to making informed decisions about the need for immunization services and, in some instances, other primary health care services. HBRs extend the relationship between the health care worker and client or caregiver beyond an individual health encounter by improving caregiver understanding and expectations about health services. Of course, to fulfill these functional roles, certain conditions must be satisfied. For example, HBRs must be available in the right place, at the right time, and in the right quantity to avoid stock-outs.^1^ They must be valued and retained by caregivers, and health care workers must request them, reference them, and ensure they are legibly completed and up-to-date. Importantly, HBRs are a document rightly due of all newborns and their caregivers as part of national promises made by signatories to the Convention on the Rights of the Child (Articles 3 and 24)^2^ to protect children's health through primary health care and engagement of caregivers in making decisions about the health care (and protection from vaccine-preventable disease) of their children.

BOX 1 **Functional Roles and Requisite Conditions of Home-Based Records**

**Functional Roles**

- Home-based records (HBRs) serve as a tool for documenting vaccinations and other primary care services, particularly during childhood but increasingly across the life course, in a standardized manner.
- When appropriately completed and referenced, HBRs provide necessary information for frontline clinical decision making that may ultimately improve continuity of care and reduce inefficiencies (for example, missed opportunities for vaccination and instances of unnecessary extra vaccination).
- HBRs complement facility-based record systems and serve as a verified surrogate in the absence of functioning facility-based record systems.
- HBRs help stimulate demand for vaccination services by raising caregivers' awareness of the benefits of vaccines, the recommended vaccination schedule, and the date of the child's next vaccination visit.
- HBRs serve as a prompt to initiate a discussion between health care workers and caregivers about the importance of immunization during a health encounter at a facility or during an outreach session.
- HBRs serve as a source of documented evidence of vaccination history that is important for public health monitoring through small-scale community-based rapid coverage assessments and larger population-based cluster coverage surveys.

**Requisite Conditions**

- HBRs are designed alongside end-users to meet their needs and to facilitate appropriate understanding and use.
- HBRs are printed on durable material and/or made available with protective covers to help them withstand exposure to harsh environments.
- HBRs are printed in sufficient quantities and appropriately distributed to avoid stock-outs.
- HBRs are made available to caregivers (ideally, free of charge) with appropriate counseling about the importance of the document and to keep the document safe from harm.
- HBRs are brought by caregivers to each health encounter, regardless of reason for the visit.
- HBRs are requested, referenced, and updated in legible handwriting by health care workers at each health encounter.

In September 2018, the World Health Organization (WHO) published the *WHO Recommendations on Home-Based Records for Maternal, Newborn and Child Health*.^3^ The guidelines were the result of a 2-year process that identified, reviewed, graded, and discussed available evidence on the potential benefits of HBRs for maternal, newborn, and child health (MNCH) outcomes. While the 2018 HBR guidelines are not the first issued by WHO to countries,^4^ these guidelines have been produced following widely accepted, transparent, and systematic methods,^5^ which combines, in a collaborative process, research evidence from systematic reviews and other types of evidence to produce qualified recommendations. Having studied HBRs from the perspective of immunization service delivery since 2010 (DWB) and engaged with targeted learning on HBR design, availability, and use from 2016 to 2018 (LS),^6^ we are encouraged by these efforts and the opportunity the guidelines present to reflect on current patterns of HBR use. We are equally excited to see how the community responds in defining an agenda for future research and action.

The findings and recommendations set forth in the 2018 HBR guidelines underscore several themes. First, despite a relative lack of robust evidence, the HBR Guideline Development Group concluded that HBRs are generally a feasible tool within MNCH programs; HBRs are particularly valued and used as a critical component of immunization service delivery.^7^ Second, much of the HBR research was conducted prior to the year 2000 or in high-income countries. As such, populations in low- and middle-income countries, which may benefit most from HBRs as a primary form of documented evidence for basic health care, are disproportionately underrepresented. Finally, HBRs have evolved over time^8^ in form and function—from simple cards issued in the 1800s to document proof of vaccination against smallpox^9^^,^^10^ to the comprehensive MNCH handbooks (rich with public health messaging as well as clinical and public health recording areas) used by some countries today. The variety of HBR designs and formats^11^ mark this evolution and reflect expanded MNCH knowledge. For example, growth monitoring charts first appeared in HBRs during the 1960s and 1970s—supporting the importance of infant and young child feeding with periodic growth monitoring.^12^ More recently, HBRs have been used to deliver information about bed nets, handwashing, management of diarrhea, and other health topics to caregivers. In some instances, HBR content has reflected multilateral and bilateral donor development aid and assistance investments and influence with agencies lobbying by including HIV status, testing, and treatment content in HBRs and encouraging the use of MNCH handbooks over other HBR formats.

Home-based records have evolved from simple cards to document proof of vaccination to comprehensive handbooks.

Rarely have the evolutionary changes in HBR content and form been accompanied by evaluations aimed at understanding the impact of changes on HBR function and MNCH outcomes. Do HBR design and use patterns satisfy the desired operational functions and end-user values, comprehension, and needs? In most instances, we don't know. The 2018 HBR guidelines noted numerous, persistent knowledge gaps. Identifying these evidence gaps is important to focus future primary research, systematic reviews, and the guideline development process. It is also noteworthy because recognizing the voids helps us to consider context and chart a course forward.

For example, the utility of growth monitoring and its relevance to the promotion of child health during the first years of life are unquestioned and the technical growth standards that underlie growth monitoring charts have been rigorously developed,^13^ despite the paucity of robust evidence on its effects.^14^ Unfortunately, growth monitoring charts found on HBRs around the world are complicated in their design—the format does not support health care worker use or caregiver understanding nor are they reinforced in practice. As a result, suboptimal use of growth monitoring charts by health care workers was recognized in the mid-1980s^15^^,^^16^ and areas for recording growth on HBRs still often remain blank.^17^ Evidence shows that health care workers do weigh and measure children; however, the measurements are often not recorded in the growth monitoring chart. Some workers adopt alternative approaches to record these measurements for monitoring purposes (such as writing the measurements in a notes section), but oftentimes this information is lost. Consequently, the valuable HBR tool is incomplete with unused colored charts, adding pages to the HBR and driving up the cost of the document.

Another concern is a shift toward comprehensive MNCH handbooks, which have become just that—hefty volumes filled with text and graphic health messages across numerous intervention areas. Despite this potentially massive investment, it remains unclear whether holistic health handbooks are useful or appropriate. In some HBRs, readability is often misaligned with the literacy levels of caregivers and, in some cases, health care workers. Additionally, graphics may not be understood if images are not vetted with a generalizable audience of end-users to ensure messages are clear. In countries challenged by scarce health resources or where community literacy levels are improving yet remain low, comprehensive MNCH handbooks may not be prudent. Moreover, the higher costs of these records^18^ may contribute to challenges with HBR stock availability.^1^^,^^6^ Research is needed to test whether MNCH handbooks are superior to alternatives, and if so, under what conditions.

Despite the potentially massive investment, it remains unclear whether holistic health handbooks, as an alternative to simple cards, are useful or appropriate.

With the introduction of health handbooks, the breadth of HBR content has also expanded to include clinical gynecologic and obstetric histories for women and detailed examination recording tables and sometimes even lab results for newborns—information that is clinically important but potentially better maintained in a clinical, facility record. Although HBRs may be well suited to complement suboptimal use of facility-based record systems, this does not ensure that information is better kept or more accessible in HBRs based on our observations. Similarly, documentation of childhood oral health assessments and treatments is important; however, we question whether such content belongs in an HBR, particularly in countries where access to dental services remains suboptimal or where dental problems in childhood are not so prevalent. And instruction on the use of insecticide-treated bed nets is undoubtedly important in preventing malaria; however, it remains unclear whether individuals who maintain an HBR with these instructions are any more, or less, likely to appropriately use a bed net than those without the HBR instruction. In the end, many HBR content areas are unreferenced (despite lengthening the document and adding to production and printing costs of the HBR) and often unused—indirectly sending an adverse message to caregivers that the content is not important.

Many content areas in the home-based record are unreferenced and often unused.

Given that HBRs have evolved without appropriate evaluation of content and design, it is no surprise that the WHO HBR Guideline Development Group was unable to recommend any one HBR format over another. In fact, the quality of the available evidence led the group to assign very low or low certainty evidence scores to most of the few identified HBR research studies. This is certainly a disappointment to those who believe, quite strongly, that MNCH handbooks are superior to vaccination-only cards or vaccination-plus-growth monitoring cards because they may reduce the need for multiple records^19^ or support improved continuity of care.^20^ While this may be true, or true for some populations under certain circumstances, at this point, we simply do not know. No appropriate research studies have examined this very important question.

The systematic review supporting the HBR Guideline Development Group thinking did not identify any studies that explored whether HBR use was associated with more equitable MNCH outcomes, nor did it find any reports of undesirable effects of HBRs, although care must be taken with such a result since the *absence* of evidence about undesirable effects is not the same as evidence of the *lack* of undesirable effects. Undesirable effects may include disproportionate production costs, misleading caregivers, diverting attention from some health care areas in favor of others, and misuse and confidentiality breaches, among others. Studying the potential undesirable effects of HBRs or their design features is crucial, particularly given recording of HIV testing, status, and treatment within some HBRs—a practice that is questionable from an ethical perspective and that should be reconsidered given the sensitive and potentially stigmatizing and discriminatory nature of the information.^3^

Consistent with prior reports,^21^ the 2018 HBR guidelines identified no economic evaluations of HBRs. A recent case study^22^ suggests cost savings with use of integrated HBRs but falls short of the rigorous economic evaluation needed to formulate well-informed guidelines or recommendations. While an informal, theoretical business case for investment in HBRs has been discussed,^21^ opportunities remain to demonstrate with greater rigor whether such a business case exists as well as the cost-effectiveness of different HBR designs (for example, MNCH handbook versus vaccination-plus-growth monitoring card).

We would like to see the modest momentum of the past 10 years continue. Since 2010, the revitalization of HBRs within immunization service delivery has taken several incremental steps forward and, based on observations at regional immunization program manager meetings and recent work within several countries, there is a feeling that modest improvements have been made in awareness of HBR availability and use among immunization programs. In 2011, the Bill & Melinda Gates Foundation supported the *Records for Life* contest,^23^ which reinforced the importance of user-centered HBR design. Workshops in South Asia^24^ and Africa^25^ alongside a JSI Inc. learning project^6^ further explored the user-centered approach and led to redesigned HBRs in Afghanistan, Cameroon, the Democratic Republic of the Congo, Ethiopia, India, Nepal, and Zimbabwe. Now, after the release of the 2018 HBR guidelines, we ask, what lies ahead for the HBR agenda?

An expansive program of work is necessary to fill the gaps that have been identified in our understanding of HBR design, function, and implementation (Box 2): a combination of public health research, rapid prototyping, usability testing, and other mixed-method approaches is required. As part of the process for updating guidelines, we must promote the uptake of the identified knowledge gaps, particularly in relation to questions for which no evidence was found and questions supported by only low certainty evidence. This is particularly the case with regards to questions of impact of the HBR on outcomes, the role of HBR design in the document's uptake and use, and the business case for investing in HBRs in the first place. This program of work extends far beyond any individual donor, country, or institution; it must not be taken on as a siloed program but as a part of a broader, ongoing effort to support the delivery of high-quality, universal primary health care to all people regardless of who they are or where they live. Robust research is required and possible, as some examples brilliantly show.

A mix of public health research, rapid prototyping, usability testing, and other approaches is required to fill the identified gaps in knowledge about home-based records.

BOX 2 **Research Questions Addressing Knowledge Gaps About Home-Based Records**

**Impact of home-based records on MNCH outcomes**

- Do HBRs impact MNCH outcomes, particularly in low- and middle-income countries?
- Which HBR components impact MNCH outcomes?

**Home-based record design**

- Are certain HBR formats (health handbooks, vaccination-only cards, vaccination-plus-growth cards) superior to others, and, if so, under what circumstances?
- Does using an HBR designed with durable paper or protective sleeves improve the likelihood that the HBR will be retained by a caregiver through the first 5 years of a child's life?
- Do certain public health messaging topics or presentations in an HBR influence health care-seeking behavior?

**Improving home-based record uptake and use**

- Can HBR design features improve the uptake and value of the document by caregivers and health workers?
- Are incentives an effective and sustainable strategy for improving HBR uptake and use?

**Economic evaluation of home-based records**

- Are comprehensive MNCH handbooks cost-effective as compared to vaccination-plus-growth monitoring records or vaccination-only HBRs?
- Is using HBRs for delivering public health messages cost-effective?
- Should countries consider using paid advertising within HBRs to help finance HBR costs?

**Understanding potential ethical concerns of home-based records**

- Is it ethical to include HIV testing, status, and treatment recording fields within HBRs?
- How can we determine the potential harm of HBR content or design from the perspective of end-users?

**Home-based record systems challenges**

- Does bundling HBRs with other vaccine delivery supplies, both in terms of forecasting and procurement, reduce HBR stock-outs?
- Is using an HBR coordinating committee an effective strategy for overcoming the challenges of fragmented oversight in countries where multiple ministry departments maintain content ownership of the HBR?
- What opportunities exist for regional market shaping to reduce the costs of durable paper products and printing services for HBRs in low- and middle-income settings?

Knowledge gaps noted above are informed, in part, by Chapter 4 and Chapter 5 of the 2018 *WHO Recommendations on Home-Based Records for Maternal, Newborn and Child Health*.^3^

We remain hopeful that the HBR knowledge base will continue to expand. New thinking and creative, collaborative solutions are needed to address existing challenges confronting HBRs and to expand and improve the availability of documented evidence of vaccination history and other child survival interventions. We appreciate the leadership of WHO and other institutions in the work completed as of today. We also believe opportunities exist for inserting HBRs and the importance of documented evidence of vaccination (and potentially other services) within the Health Data Collaborative agenda to strengthen health information systems.^26^ With commitment, coordination, and resources, these organizations and their country partners can collectively do more to propel our knowledge of the direct and indirect roles HBRs might play in global initiatives to expand birth registration, extend the lifesaving benefits of immunization to all persons, and improve infant and young child nutrition among many other areas of maternal and child health.

## Notes

**Peer Reviewed**

**First Published Online:** March 15, 2019

**Funding:** This work received no specific grant from any funding agency in the public, commercial, or not-for-profit sectors.
